# Hermansky–Pudlak/Chediak–Higashi syndromes

**Published:** 2012-07

**Authors:** Hilary Denis Solomons

**Affiliations:** Johnnesburg, South Africa

The common denominator in both of these conditions is albinism. Hermansky–Pudlak syndrome affects the platelets and patients have a tendency to bleed.1

Chediak–Higashi syndrome affects the leukocytes, results in immune disorders and causes intracytoplasmic inclusions. These latter patients are prone to malignant lymphomas as the immune system is involved.

Hermansky–Pudlak symptoms occur due to defects in the melanosomes and the disease affects the lysosomal organelles in the cells, especially the platelet-dense granules. For this reason these patients have a haemorrhagic tendency.

Patients with Chediak–Higashi syndrome usually die at an early age. The disease also affects the lysosomal organelles.2

Chediak–Higashi syndrome is an autosomal recessive disorder, as is Hermansky–Pudlak syndrome.3 Subtypes of Hermansky–Pudlak disease exist. Chromosomes 3, 5 and 10 are involved. Hermansky–Pudlak is seen predominantly in Puerto Ricans but is also found in the Swiss Alps.

In Chediak–Higashi disease, eight known gene allele defects are found, natural killer cells are deficient and the immune system is involved, predisposing patients to lymphomas. In both disorders hair, skin and eye colour are deficient, making albinism the common factor.3

It can therefore be concluded that both Hermansky–Pudlak and Chediak–Higashi syndromes affect the platelets and white cells, namely the haematological system.
